# Should the Nurse Articulate?

**Published:** 1918-12-07

**Authors:** 


### Should the Nurse Articulate?

*To the Editor of* The Hospital.

Sir,—Should nurses articulate? Well, this one is going to try, although the attempt may be a poor one. I have carefully read all "Ierne's" letters. I don't care a scrap if "Ierne" be a man or a woman. If a man, well, he possesses a most uncommon amount of sense for a man; if a woman, she is a brick and deserves support. First, let me say how much I agree with the letter from "Chrysanthemum." It is slavery that makes one turn against nursing; give us better conditions, better food, higher wages, and shorter hours, and then I am quite sure that nurses will be happier and better right through. It is becoming an accepted fact that nurses are shamefully underpaid. We are told that nursing is the noblest profession on God's earth, and we are paid the wage of a scullerymaid. We don't want flattery. We want decent wages, so that we can provide for old age. We don't want pensions: we want to be able to provide for ourselves. In one of the large military hospitals of the North the following was posted up:

"The Director-General wishes it to be known in all military hospitals in the Northern Command how much he appreciates the unselfish devotion to their duty of the members of the Nursing Services at this time of emergency. He is aware that they are being very much overworked and regrets that, in spite of all efforts which have been and are being made to procure more nurses, it is quite impossible, owing to the widespread epidemic of influenza, to send the necessary help. The admirable spirit of devotion to duty of all ranks of the Nursing Service will be brought to the notice of the Nursing Board of the Army Council."

This is very nice, but compliments don't fill pockets. The wages of Army nurses are the same now as in 1914. Prices are not the same. The War Office promised to their nurses a war bonus of £7 10s. per annum. In this particular hospital it has never been paid. After reading the above notice the nurses thought it a good time to ask for the bonus. Another notice appeared to the effect that the war bonus was not a bonus but a gratuity, and if we left before the hospital was closed—well, we would not get it; in fact, after reading the notice one was left in doubt if the gratuity (?) would ever be paid at all.

This is the way the nation treats its nurses. Where would England have been during the war and the present epidemic had it not been for the nurses? How many lives have they saved? I think it is quite time the College of Nursing woke up and tried to fulfil their promises; if they cannot do anything, then let us try something else.

Merely a Staff Nurse in the North.

